# Extensive Cutaneous T-Cell Lymphoma of the Feet Treated with High-Dose-Rate Brachytherapy and External Beam Radiation

**DOI:** 10.1155/2018/5610925

**Published:** 2018-08-07

**Authors:** Joy Tao, Courtney Hentz, Michael L. Mysz, Issra Rashed, David Eilers, James Swan, Rebecca Tung, Bahman Emami

**Affiliations:** ^1^Stritch School of Medicine, Loyola University Chicago, Chicago, IL, USA; ^2^Department of Radiation Oncology, Stritch School of Medicine, Loyola University Chicago, Chicago, IL, USA; ^3^Section of Dermatology, Hines Veterans Affairs Hospital, Hines, IL, USA; ^4^Division of Dermatology, Department of Medicine, Stritch School of Medicine, Loyola University Chicago, Chicago, IL, USA

## Abstract

Cutaneous T-cell lymphoma (CTCL) is a chronic, debilitating disease that has a severe impact on quality of life. We present a patient with multiple CTCL lesions on the bilateral feet, which impaired his ability to ambulate. His lesions on both feet were successfully treated with a total of 8 Gy in two fractions via high-dose-rate surface brachytherapy using the Freiburg Flap applicator. The deeper aspects of the bulkier lesions on the left foot were boosted with electron beam therapy. The radiation therapy was well tolerated, and the patient was able to regain his mobility after completing radiation therapy. To our knowledge, there are few reports utilizing brachytherapy in treating CTCL. Our case describes treatment of larger, more extensive CTCL lesions than previously reported.

## 1. Introduction

Cutaneous T-cell lymphoma (CTCL) is a chronic, debilitating condition that accounts for approximately 4% of all non-Hodgkin lymphomas, with mycosis fungoides being the most common type [1]. Current strategies and goals of CTCL treatments include alleviation of symptoms, control of local disease, and improvement in quality of life [2]. We report on a patient with multiple CTCL lesions refractory to standard therapies who received a combination of external beam radiation therapy (EBRT) and high-dose-rate (HDR) brachytherapy for organ preservation and to control his painful disease.

## 2. Case

A 69-year-old male presented to dermatology clinic with stage T2b mycosis fungoides, diagnosed two years prior, which manifested as a persistent, chronic rash involving both feet, and, to a lesser extent, other sites of his body. The lesions on his feet were painful and pruritic, limiting his ability to wear shoes and ambulate for the past two years. His disease showed little to no response to numerous topical agents including topical nitrogen mustard, imiquimod, clobetasol, vinegar soaks, PUVA soaks, amoxicillin, and doxycycline. Per the patient, consideration was made for amputation of the left foot below the ankle, which he refused. Subsequently, he was referred to radiation oncology.

**Figure 1:**
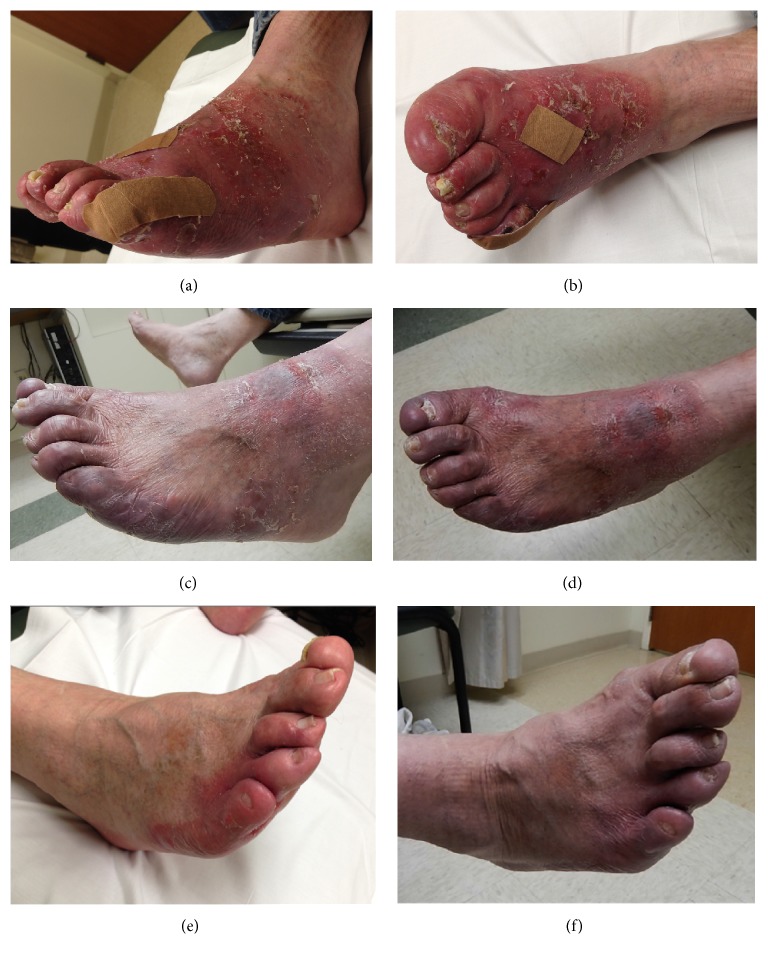
(a) and (b) Left foot at presentation. (c) and (d) Left foot at follow-up 11 months later with a new 3-4 cm circular lesion that developed just proximal to the irradiated area. (e) Right foot at presentation. (f) Right foot at follow-up 11 months later.

Physical exam revealed tender, confluent, erythematous, and desquamated patches on the skin extending from the dorsal and ventral surfaces of his left foot to the ankle (Figures 1(a) and 1(b)). His right foot had smaller, erythematous patches proximal to the 4^th^ and 5^th^ digits extending between the digits (Figure 1(e)). He was recommended surface HDR brachytherapy to his symptomatic lesions. The patient agreed to begin radiation therapy first to his most prominent and painful lesions on his left foot and undergo subsequent treatments for his other lesions if results warranted. A preliminary scan of the left foot showed diffuse involvement with some dorsal lesions > 5 mm in thickness. The patient was recommended 8 Gy in 2 fractions of superficial HDR brachytherapy to the entire affected area of his left foot using the Freiburg Flap (FF) applicator (Elekta AB, Stockholm, Sweden) followed by 20 Gy in 10 fractions of 6 MeV external beam electron treatments to the bulky dorsal lesions.

The FF applicator consists of a planar array of 1 cm diameter silicone spheres with longitudinal channels for insertion of treatment catheters and flexible connections laterally which enable the FF to conform to highly curved and irregular surfaces. The FF is often affixed to a thermoplastic mesh (TM), commonly used in radiation therapy, to maintain a reproducible orientation relative to the patient's anatomy.

**Figure 2:**
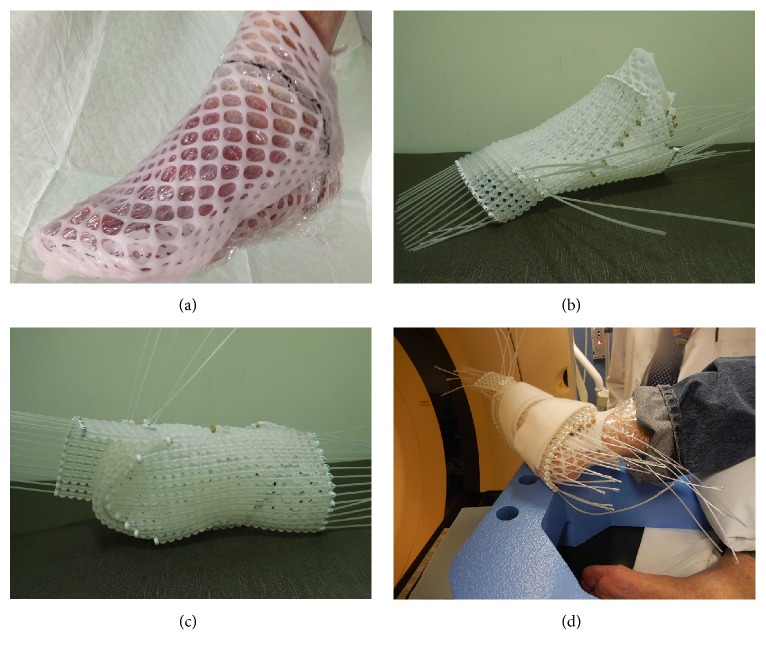
(a) Two-part thermoplastic mesh encompassing the left foot with proximal border of treatment area outlined in black. (b) Complete Freiburg device consisting of a total of 39 catheters. (c) Plantar aspect of the Freiburg device. (d) Positioning of the left foot for CT scan and treatment.

In preparation for this patient's left foot HDR treatment, two pieces of TM material (Extremity EMRT-8430, Bionix Inc., Toledo, OH) were heated and formed around the patient's left foot consisting of a dorsal part and corresponding plantar portion. This two-part, clam shell construction allowed the entire foot to be tightly enveloped by TM yet provided ease of ingress and egress (Figure 2(a)). The FF was attached to the TM with dental floss interwoven between the FF beads and through the TM struts. This TM design and position of the FF catheters enabled the Ir-192 HDR source to travel in close proximity to the cutaneous tissue to be treated. A total of 39 catheters were required to encompass the entire treatment area of his left foot (Figures 2(b) and 2(c)).

**Figure 3:**
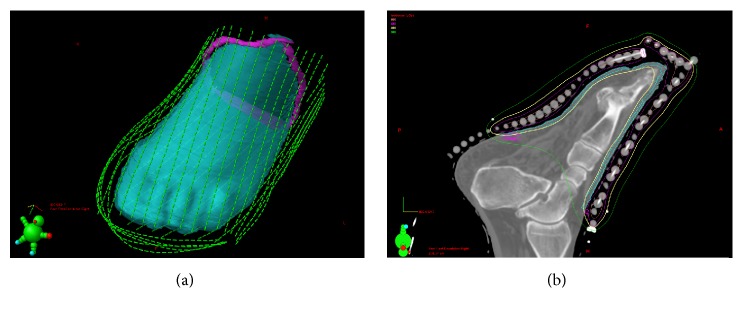
(a) Left foot treatment plan: 3D view of source dwell positions. (b) Left foot treatment plan: cutaneous planning treatment volume is shown in cyan, and the delivery of a highly conformal 4 Gy of radiation is shown in yellow isodose line.

For treatment planning, a CT scan was performed with the FF device firmly affixed to the patient's foot and a thin metal marker wire attached to the TM to delineate the intended treatment borders (Figure 2(d)). The images were imported into Eclipse (Varian Medical Systems, Palo Alto, Ca). The planning treatment volume (PTV) outlined or ‘contoured' on the CT images consisted of a 1 cm proximal margin and all cutaneous surface tissue below the level of the marker wire to a depth of 3 mm. The path of each FF catheter was identified in three dimensions, and dwell positions for the HDR source were selected in step increments of 5 mm (Figure 3(a)). The dwell times of these source positions were adjusted to provide uniform coverage of 4 Gy to the peripheral margins of the PTV (Figure 3(b)). Overall, the left foot treatment plan involved 1326 active sources across 39 catheters, with a total dwell time of 1407 seconds (for a 10 Ci source). Two fractions of 4 Gy were delivered every other day during one week.

The treatment was well tolerated with some mild radiation-related edema and associated left foot pain that was managed conservatively and resolved within a week of completing treatment. At one-week follow-up, his lesions were regressing with significant improvement in pain, scaling, and erythema. Four months later, the deeper aspect of the gross tumor lesions involving the left foot was boosted with 20 Gy in 10 daily fractions using 6 MeV external beam electrons. Additionally, the same brachytherapy process without EBRT was subsequently followed for the patient's right foot, which responded well to HDR brachytherapy.

At each short-term follow-up (≤ 6 weeks) after completing his HDR brachytherapy, he reported a rewarding response with improvement of disease-related erythema, pain, and swelling in all treated areas, with near resolution of treatment related hyperpigmentation (Figures 1(c), 1(d), and 1(f)). Both feet were still in remission at his most recent follow-up 21 months and 19 months after completing his left and right foot treatments, respectively. Additionally, he was ambulating and wearing shoes, which he was unable to do at presentation due to his painful lesions. He did develop a new 3-4 cm mildly erythematous, circular lesion on the dorsal surface of his left foot just proximal to the irradiated area, which was treated and controlled with topical steroids (Figures 1(c) and 1(d)).

## 3. Discussion

CTCL lesions are extremely radiosensitive, with some reports suggesting that low-dose radiation therapy can achieve successful results (Table 1) while allowing for fewer clinic visits, decreased costs, less toxicity to the skin, and reirradiation in the future due to symptom relapse [3, 4]. Neelis et al. utilized low-dose external beam radiotherapy to treat mycosis fungoides lesions refractory to PUVA and topical steroids. They reported that 65 lesions in 24 patients treated with 8 Gy in two fractions had a complete response rate of 92% with no skin toxicities noted [3]. Thomas et al. found a complete response rate of approximately 94.4% among 58 patients with CTCL treated with a single fraction of radiation therapy, the majority between 7 and 8 Gy, using either photons or electrons. The mean follow-up time was 41.3 months, and no significant long-term side effects were observed [4]. Low-dose total skin electron beam therapy has also shown satisfactory results with a good side effect profile for patients with more diffuse skin disease [5–8].

Brachytherapy is a technique in which a radioactive source is placed directly into or adjacent to target lesions via implants or surface applicators [9, 10]. One such applicator is the Freiburg Flap, which is designed to allow the HDR Ir-192 source to travel approximately 5 mm from the skin surface. This method is noninvasive and ideal for delivering tumoricidal doses of radiotherapy to superficial lesions while limiting unfavorable delivery of radiation to healthy tissues due to rapid dose fall-off at the periphery of the lesions. This is especially desirable when treating anatomic sites that are near tissues vulnerable to irradiation or that present significant cosmetic challenges to surgical excision such as the scalp, face, and hands [9]. With use of custom molds such as the FF, this technique also permits delivery of a uniform radiation dose to uneven surfaces, which was advantageous in the treatment of our patient's feet, whereas EBRT is often utilized for deeper lesions and on flat surfaces [11]. Some lesions on the dorsal aspect of our patient's left foot were greater than 5 mm in thickness, and the EBRT was added to treat the deeper, bulkier parts of the tumor, whereas only the FF brachytherapy was required for the thinner patches on the right foot. Therefore, treatment with the FF alone is sufficient for superficial lesions contained within 5 mm of the skin surface.

While brachytherapy treatments for nonmelanoma skin cancers have been extensively studied and found to have high local control rates and excellent cosmetic outcomes [9, 10, 12–14], there are few reports utilizing brachytherapy for CTCL. DeSimone et al. reported on 10 patients with facial mycosis fungoides lesions that were successfully treated with HDR brachytherapy doses of 8 Gy in 2 fractions of 4 Gy. There were no recurrences in the median 6-month follow-up period [15]. Goddard et al. presented a case series utilizing HDR brachytherapy for the treatment of acral CTCL skin lesions on six patients with eight lesions also treated with 8 Gy in 2 fractions. They reported an 88% control rate with only one lesion recurring locally within a mean follow-up period of 15.8 months [16]. Our case describes treatment of more extensive CTCL lesions than previously reported.

The symptom burden of CTCL significantly impairs the quality of life for patients. Patients frequently experience intense pruritus, pain, cracking and bleeding skin, and associated insomnia, depression, and decreased self-esteem [17, 18]. Depending on the location of the lesions, patients may have difficulty using their hands or ambulating [16]. Treatment with radiotherapy improved our patient's symptoms and allowed him to regain his mobility and avoid possible amputation of his afflicted left foot. Our case, as well as the previous literature, suggests that HDR brachytherapy is a potentially valuable palliative, organ sparing treatment for CTCL lesions, particularly those refractory to more traditional therapies. Further studies are needed to establish treatment guidelines as well as evaluate long-term control rates and outcomes.

## Figures and Tables

**Table 1:** Local cutaneous T-cell lymphoma lesions treated with low-dose radiation therapy.

**Reference**	**Number of Patients**	**Number of Sites**	**Radiation Technique**	**Total Doses (Gy)**	**Local Recurrence Rate**	**Mean Follow-up**
Neelis et al, 2009 [3]	24	65	Electron beam	8.0 Gy in 2 fractions	5/65 (7.7%)	9.6 months
Thomas et al, 2013 [4]	58	270	Photon or electron beam	≥7.0 Gy in 1 fraction	4/270 (1.5%)	41.3 months
DeSimone et al, 2013 [15]	10	23	HDR brachytherapy	8.0 Gy in 2 fractions	0/23 (0%)	6.3 months (median)
Goddard et al, 2015 [16]	6	8	HDR brachytherapy	8.0 Gy in 2 fractions	1/8 (12.5%)	15.8 months

## Abbreviations

CTCL: Cutaneous T-cell lymphoma
Gy: Gray
HDR: High-dose-rate
EBRT: External beam radiation therapy
FF: Freiburg Flap
TM: Thermoplastic mesh
PTV: Planning treatment volume.
